# Responding to the COVID-19 outbreak as a therapeutic community in a forensic psychiatric ward in Japan–A reconsideration of the role of therapeutic community in disasters

**DOI:** 10.3389/fpsyt.2022.577969

**Published:** 2022-08-10

**Authors:** Hiroko Kashiwagi, Kayo Kume, Koji Takeda, Taiki Ueshima, Osamu Asaumi, Mayu Omori, Naotsugu Hirabayashi

**Affiliations:** Department of Forensic Psychiatry, National Center Hospital, National Center of Neurology and Psychiatry, Kodaira, Japan

**Keywords:** Japan, COVID-19, therapeutic community, forensic psychiatry, MTSA, disaster

## Abstract

The global impact of COVID-19 outbreak on psychiatric hospitals and prisons is unfathomable and unprecedented, and information is needed on how best to mitigate widespread infection whilst safeguarding the community's well-being. This study reports on how the staff and patients in a forensic psychiatric ward in Japan worked together during the COVID-19 outbreak as a “therapeutic community.” The “Non-Three Cs” Karaoke Project, with infection prevention guidelines designed by inpatients, was safely conducted and its humor released the staff and patients' anxiety and tension. Through these discussions, the patients and staff gained a better understanding of viruses, transmission routes, countermeasures, and coping with stress. The study highlights the importance of disclosing information to inpatients, conducting open discussions, and involving patients in the prevention and management of infectious diseases. This report is the world's first report showing a concrete example of the therapeutic community's significance during the COVID-19 outbreak. It is an experience that offers an opportunity to reconsider the significance of the therapeutic community, in which patients are seen as a presence that brings change, strength, growth, and creativity into the therapeutic setting. We believe that such an approach in a future disaster would lead to an increase in the patients' problem-solving ability, and recovery and autonomy after discharge could be promoted. A shared difficult situation can be an opportunity to build a therapeutic alliance and make a difference.

## Introduction

The impact of the coronavirus disease (COVID-19) outbreak on psychiatric hospitals and prisons around the world is unfathomable (1, 2). Riots over COVID-19 rules have taken place in various prisons (3). We report how the therapeutic community in a forensic psychiatric ward in Japan worked during this time of crisis.

Clusters have occurred in several psychiatric hospitals in Japan, and tension between hospital staff continues to increase: the increase in tension is consistent with the Medical Treatment and Supervision Act (MTSA) wards in Japan which aim to provide intensive psychiatric treatment for individuals who commit serious offenses, such as homicide, robbery, bodily injury, arson, rape, and indecent assault in a state of insanity or diminished responsibility as well, due to concerns for ward structure prone to clustering, some patients who have difficulty understanding, restrictions on outings, overnight stays, and leisure activities, as well as a reduction in staff due to telework. They leverage providing support for inpatients' return to society. MTSA wards have been operating since 2005, with more than 80% of inpatients being diagnosed with schizophrenia (4).

MTSA wards are based on British Democratic Therapeutic Community principles (5–7). In 1938, Maxwell Jones, who was treating neurotic patients due to war experiences that stem from the battlefield, stated that “there was little reaction from the lecture, but the discussion and lateral connections between the patients worked therapeutically” (5). Community meetings involving patients, nurses, and doctors were held to discuss everyday issues and ward management (5, 8), and they developed interventions to help patients cope with and avoid stress (5, 9). In 1946, Tom Main first introduced the concept of “therapeutic community.” Furthermore, Stevens concluded that these series of achievements confirmed the potential of therapeutic community and transformed a psychiatrist's presence to a modest technician among patients and from a supervising role to a growth-promoting role (5, 10). Thus, daily short meetings and weekly long meetings are now held to discuss everyday issues in each unit (the MTSA ward comprises an acute, recovery, and rehabilitation stage), and a monthly general meeting for all inpatients (up to 33 people can be hospitalized in one ward) and multi-disciplinary staff meeting is also held to discuss issues and activity plans.

When the therapeutic community is mentioned in the context of a disaster, it is often referred to with regard to the role of sharing painful experiences and feelings among the victims (11–13), for example, the Idobata-Nagaya community housing after Fukushima's triple disaster in the 2011 Great East Japan Earthquake (14). There are many natural disasters in Japan, and hospital facilities are often damaged by major earthquakes, typhoons, and floods, amongst others, and electricity and water supply may be disrupted (15). However, there are few reports regarding the role of therapeutic community in facilities, including the forensic inpatient facility at the time of disaster.

The effectiveness of therapeutic communities in forensic settings has also been reported on. Therapeutic communities have improved outcomes in patients with severe mental illnesses such as schizophrenia (16), while also reportedly contributing to the prevention of anger and self-harm (17). Therapeutic communities also reportedly reduce reincarceration in drug using offenders with co-occurring mental illness (18). On the other hand, even in organizations that incorporate therapeutic communities, staff may provoke anger in patients (17). Reacting to patient anger in a non-instinctive way in the therapeutic community can prevent a vicious cycle; moreover, improved skills among staff may reduce their stress and burnout (17).

We hypothesize that the therapeutic community model was effective in our ward because it centers on the idea of respect for the patient's opinion, which helps ensure that patients are provided with reassurance. When patients take on new roles and participate in the management of the ward on the basis of shared decision-making and therapeutic alliances between staff and patients, they experience being useful to others. This can enhance their ability to examine reality and improve their self-efficacy.

In infection control, it is important to provide accurate information and involve patients in infection prevention measures (1, 19). In addition, everyone is anxious about getting infected and the risk of being forced to change their lifestyles produces a considerable amount of stress. Considering that the concept of Democratic Therapeutic community comprises of social learning, permissiveness, and agency (20–22), and that this also is valid for the therapeutic community in the forensic setting (23), there should also be room to consider the significance of therapeutic community during a pandemic, such as the COVID-19 outbreak, in the forensic psychiatric wards.

The Japanese government declared a state of emergency on April 8, 2020, related to the spread of COVID-19. The government recommended avoiding three specific situations to prevent virus spread: closed spaces with poor ventilation (*Mitsupei*), crowed places (*Mitsushu*), and close-contact settings with close-range conversations (*Mitsusetsu*): these are known as the “Three Cs” (“3 *Mitsu”* in Japanese) (24).

The objectives of this case report are as follows: we present a retrospective report of how a therapeutic community functioned in a Japanese forensic psychiatric ward (MTSA) in the midst of heightened tension and anxiety in the ward due to the spread of COVID-19 infection. We show how the therapeutic community functioned effectively and what changes occurred in the patient and his environment, depending on the type of patients involved, the personal philosophy and skills of the staff, and how they intervened. It also seeks to promote research and reporting on the significance of therapeutic communities in the event of such disasters.

## Case study

### Methods

A charge nurse with years of experience, with communication skills based on cognitive behavioral therapy, motivational interviewing, patient-centered care, and knowledge of good therapeutic alliances, worked with the patient on the project. The physician in charge and the head nurse supervised both the charge nurse and the project. All of them were trained in basic communication skills, including cognitive behavioral therapy, motivational interviewing, de-escalation, shared decision making, and had basic knowledge of case formulation and trauma informed care. Moreover, all had a basic knowledge of the values and principles of therapeutic communities, therapeutic alliances, and patient-centered care. They were not required to have any specific certification, but they did attend annual training sessions that ranged from a few hours to several days. In addition, the charge nurse and physician had experience as Wellness Recovery Action Plan (WRAP) facilitators. The name of the project was conceived by the patient who took part in the core project. Knowledge of COVID-19 infection prevention measures was shared using materials. Together the patient and the charge nurse developed a written plan and presented it at a unit and ward-wide meeting attended by all inpatients. The project itself (leisure time activities) was attended by patients approved by the multidisciplinary team in charge. Usually, patients with acute symptoms such as psychomotor agitation or significant hallucinatory or delusional states did not participate. For the patient who took part in the core project, the ICF (International Classification of Functioning, Disability and Health) (25) functioning and ICF environmental factors and GAF (Global Assessment of Functioning) (26) were assessed every 6 months by the multidisciplinary team in charge. The charge nurse, charge occupational therapist, and charge social worker assessed ICF functioning, while the social worker assessed ICF environmental factors and the charge physician assessed GAF. Data were taken from report sheets submitted to the court every 6 months after admission for application for continued hospitalization or discharge.

### Case presentation

Inpatients in MTSA wards have been informed about the infection status in our institution and the surrounding community, as well as the countermeasures taken. Treatment programs, including leisure activities, have been limited, for example, promoting staff telework has reduced time spent in psychological interviews. Opportunities to go out, overnight stays, and meetings with family are limited. Care program meetings with community supporters are also limited, and many inpatients are not being discharged.

An inpatient with schizophrenia who manages a patient-centered activity group expressed that he wished to resume karaoke, a leisure activity popular in Japanese culture. This hinders COVID-19 prevention, since it involves gatherings in enclosed spaces, singing, and sharing a microphone. Thus, some nurses voiced disagreement. During a monthly general meeting, he suggested resuming karaoke sessions, stating “We cannot go out, stay overnight, or meet with our families; we are unable to coordinate discharge. Everyone is stressed out in the midst of this crisis, and in order to relieve stress, it is necessary to resume karaoke, which is the most popular program in the leisure activity group.” Several inpatients agreed with the suggestion. Accordingly, everyone decided to consider ways to safely carry out karaoke, resulting in the “Non-Three Cs (Non-3 *Mitsu*) Karaoke Project.”

The main inpatient engaged in this project had schizophrenia and was receiving medication with clozapine, was in the rehabilitation phase, and was preparing for discharge from the hospital. He was considered to have recovered his basic capacity to make decisions. His full IQ (Intelligence Quotient), as measured by the Wechsler Adult Intelligence Scale 3rd edition, was 71. His scores for ICF functioning and environmental factors and GAF before the project began are shown in Tables 1–3. He worked with his charge nurse to develop a written proposal for the Karaoke Project. During the proposal development phase, the shared goals of the patients and the staff were clearly defined as follows: to prevent infection, to relieve stress by singing songs, and to make everyone feel safe. The patient's good behavior and ideas were encouraged. The proposal was presented at several meetings of patients and staff, drew feedback from the participants, and was revised repeatedly.

**Table 1:** Change of Scale on the ICF functioning.

**ICF functioning**		**Scale**		
		**9 months before the project**	**3 months before the project**	**3 months after the project**
Self-care	Ensuring one's physical comfort	1	1	1
	Managing diet and fitness	2	2	2
	Maintaining one's health	2	2	2
	Preparing meals	1	1	1
	Doing housework	2	2	2
Basic interpersonal interactions	Respect and warmth	0	0	0
	Appreciation	0	0	0
	Tolerance	1	1	1
	Criticism	2	0	0
	Social cues	2	1	1
	Physical contact	0	1	1
Complex interpersonal interactions	Forming relationships	1	1	1
	Terminating relationships	1	1	1
	Regulating behaviors within interactions	2	1	1
	Interacting according to social rules	2	1	1
	Maintaining social space	1	1	1
Carrying out a daily routine	Managing a daily routine	2	1	1
	Completing a daily routine	1	1	1
	Managing one's own activity level	1	1	1
Handling stress and other	Handling responsibilities	1	1	1
psychological demands	Handling stress	2	1	1
	Handling crisis	3	8	8
Economic life	Basic economic transactions	2	2	1
	Complex economic transactions	3	3	2
	Economic self-sufficiency	4	4	4

*The following scores are used to evaluate the execution of the current life situation*.

*Scale: 0: “Fully capable (0–4% disability, independent)” 1: “Generally able (5–24% impairment, requiring supervision)” 2: “Somewhat able (25–49% disability, sometimes requiring instruction, assistance, or intervention)” 3: “Can hardly do it (50–95% disability, often requiring instruction, assistance, or intervention)” 4: “Cannot do it at all (96–100% disability, needs constant assistance)” 8: “Not specified” 9: “Not applicable”*.

*ICF, International Classification of Functioning, Disability and Health*.

**Table 2:** Change of Scale on the ICF environmental factors.

**ICF environmental factors**		**Scale**		
		**9 months before the project**	**3 months before the project**	**3 months after the project**
Products and technology	Prescription medications, personal vehicles, a home, assets, etc.	2	3	0
Natural environment and human-made changes to environment	Convenience of hospital visits, local customs, etc.	2	4	0
Support and relationships	Quantitative evaluation of human support from family members, acquaintances, medical and social workers, etc.	2	4	1
Attitudes	Qualitative assessment of the human environment, including attitudes of family and acquaintances, therapists, and the community in which they live	2	4	1
Services, systems and policies	Availability of medical welfare system, etc.	2	2	0

*Note the specifics of whether environmental factors are working in a facilitative or inhibitory manner. This information will be used to formulate an intervention policy*.

*Scale: 0: “Facilitating” 1: “Somewhat facilitating” 2: “Neither” 3: “Somewhat inhibiting” 4: “Inhibiting.”*

*ICF, International Classification of Functioning, Disability and Health*.

**Table 3:** Change of score on the GAF.

	**9 months before the project**	**3 months before the project**	**3 months after the project**
GAF score	45	50	55

*GAF, Global Assessment of Functioning*.

At each unit's patient meeting, a discussion on safely conducting karaoke sessions was held, and several opinions were expressed. Inpatients and staff refreshed their knowledge on COVID-19 prevention: transmission routes, proper handwashing, disinfection, and avoiding the Three Cs. Finally, the patient presented the completed proposal at the general meeting, and the following guidelines were suggested: (i) opening a window in a large gymnasium to ensure ventilation, (ii) allowing only up to nine participants per session, (iii) maintaining a two-meter space between individuals, (iv) wearing masks, (v) disinfecting the microphone after every use, (vi) facing the window while singing, with their backs to each other, and (vii) washing hands before and after the session. Eventually, it was decided that the karaoke should be resumed at the discretion of the inpatients after the emergency declaration had been lifted.

The inpatient who suggested the Karaoke Project had shown improvement in ICF interpersonal interactions and GAF prior to the start of the project (Table 1). Three months prior to the start of the project, all scores on the environmental factors, including support and relationships and attitude from his supporters around him, had worsened due to the spread of coronavirus, but 3 months after the start of the project, there was a marked improvement (Table 2). He was discharged from the hospital approximately 7 months after the Project began and is living a stable community life. Before his discharge, he shared with the multidisciplinary team in charge that he had been instrumental in organizing the Karaoke Project, which was helpful in the management of the ward and in relieving stress for other patients. The Karaoke Project was carried out for approximately 1 year and 6 months. More than 2 years have passed since the start of the Karaoke Project, and no COVID-19 infection has occurred among the patients in the ward.

The inpatient read the full Japanese translation of this article and confirmed that there are no mistakes. He was informed of the article's readership and provided written informed consent for publication. He stated, “I think it is important to document all this through a paper because there has been no precedent. Please watch over us warmly. I hope to work with you in the future.”

## Discussion

In measures against infectious diseases such as COVID-19, it is important to disclose accurate information (19), discuss it openly, and involve patients in infection control (1). However, decisions on countermeasures tend to become administrative and unidirectional. Information about COVID-19, infection status in the surrounding area and our facility, and infection control methods were provided to inpatients. Nursing staff sincerely listened to patients' opinions, suppressing their desire to protect, supervise, and intervene. Thus, the therapeutic community's significance was demonstrated, and through discussions on the Karaoke Project, many inpatients and staff collectively learned about viruses, infection control measures, and stress management. The Karaoke Project included some humor, which also relieved staffs' and patients' tension. In the event of a disaster, such as an earthquake or an epidemic of infectious diseases, sharing common goals for patients and staff, can turn a hard situation into an opportunity to build a therapeutic alliance. It also leads to confidence that the patient will be able to resolve any problems they encounter after being discharged.

In the midst of something like the coronavirus outbreak, a patient's suggestion of a karaoke project would normally have been instinctively and headstrongly rejected by the staff. However, even during a pandemic, the values of a therapeutic community can make sense. The atmosphere that a hospital ward therapeutic community should provide involves 1. preservation of the patient's individuality, 2. the assumption that patients are trustworthy, 3. the encouragement of good behavior, 4. the assumption that patients have considerable capacity for responsibility and initiative, and 5. activities and a proper working day for all patients (27). In this case, staff respected the patient's opinion and considered it credible, which encouraged the patient's good behavior. It should also be taken into account that this patient was considered to have recovered his basic capacity.

It is important to note that this case study has some limitations. The patient's ICF environmental factors and GAF scores improved before and after the project, but the extraneous variables that influenced them are unknown. In addition to the fact that no COVID-19 infection has occurred in this MTSA ward, we did not conduct a comparison research with other programs such as psychoeducational programs including accurate information of the COVID-19, infection route and epidemic status, and psychological impact such as anxiety. Thus, it is not clear whether such a therapeutic community approach was effective in preventing infection. Since the intensity of infection control varies depending on the area and the facility's infection status, it is necessary to carefully consider whether this method can be applied to other facilities.

Despite these limitations, this report is the world's first report showing a concrete example of the therapeutic community's significance during the COVID-19 outbreak. It is an episode that offers an opportunity to reconsider the significance of the therapeutic community in which patients are seen as a presence that brings change, strength, growth, and creativity into the therapeutic setting in case of another disaster. We believe such an approach for any disaster would lead to patients' recovery and autonomy after discharge.

## Data availability statement

The original contributions presented in the study are included in the article/supplementary material, further inquiries can be directed to the corresponding author.

## Ethics statement

Written informed consent was obtained from the individual(s) for the publication of any potentially identifiable images or data included in this article.

## Author contributions

HK treated the forensic inpatients, obtained a signed release from the patients authorizing publication, and contributed to drafting the manuscript. KK and OA contributed to therapeutic community practice with the patients. HK, KK, KT, TU, OA, MO, and NH contributed to the design and management of the case study. All authors have approved the final version of this manuscript.

## Conflict of interest

The authors declare that the research was conducted in the absence of any commercial or financial relationships that could be construed as a potential conflict of interest.

## Publisher's note

All claims expressed in this article are solely those of the authors and do not necessarily represent those of their affiliated organizations, or those of the publisher, the editors and the reviewers. Any product that may be evaluated in this article, or claim that may be made by its manufacturer, is not guaranteed or endorsed by the publisher.
